# 24-hour NIHSS may be a reliable surrogate for 90-day mRS after mechanical thrombectomy—a prospective study in anterior and posterior circulation infarctions

**DOI:** 10.3389/fneur.2025.1698534

**Published:** 2026-01-12

**Authors:** Quan Liu, Qing He, Juan Luo, Jie Yang, Guoliang Zhu

**Affiliations:** 1 Emergency Department, West China School of Medicine, Sichuan University, Sichuan University Affiliated Chengdu Second People’s Hospital, Chengdu Second People’s Hospital, Chengdu, China; 2 Clinical Laboratory, The First Affiliated Hospital of Chengdu Medical College, Chengdu, China; 3 Department of Hepatopancreatobiliary Surgery, The First Affiliated Hospital of Chengdu Medical College, Chengdu, China; 4 Department of Neurology, Sichuan Academy of Medical Sciences, Sichuan Provincial People's Hospital, University of Electronic Science and Technology of China, Chengdu, China; 5 Department of Neurology, The First Affiliated Hospital of Chengdu Medical College, Chengdu, China; 6 Department of Neurology, Nanjing First Hospital, Nanjing, China

**Keywords:** cohort study, early surrogate, ischemic stroke, mechanical thrombectomy, NIHSS

## Abstract

**Background and objective:**

The 24-h National Institutes of Health Stroke Scale (NIHSS) is a promising early surrogate for 90-day functional outcome after mechanical thrombectomy (MT). However, its predictive performance in posterior circulation infarctions (PCI) is not well established. In addition, whether this association is consistent across anterior (ACI) and PCI territories, and whether it follows a linear or non-linear pattern, remains unclear.

**Methods:**

This prospective cohort included patients from two centers (2015–2022). The primary outcome was a favorable 90-day functional outcome (The modified Rankin Scale [mRS] score ≤2). The primary analysis used multivariable logistic regression to evaluate the predictive value of the 24-h NIHSS, with results expressed as the area under the curve (AUC). Secondary analyses included testing for interaction between the 24-h NIHSS and infarct location (ACI vs. PCI) and modeling non-linear relationships using restricted cubic splines.

**Results:**

A total of 334 patients were included (ACI: 282 [84.4%], PCI: 52 [15.6%]). Median baseline and 24-h NIHSS were 16 (IQR: 11–19) and 14 (6–20), respectively. The 24-h NIHSS demonstrated the highest predictive accuracy (AUC: 0.849, 95% CI: 0.805–0.893) compared to other metrics. An optimal cutoff of ≤6 yielded a sensitivity of 94.9% and specificity of 65.8%. Critically, no significant interaction between infarct location and NIHSS metrics was found (*p* for interaction > 0.05), indicating consistent predictive performance across ACI and PCI. Furthermore, the 24-h NIHSS exhibited a non-linear association with functional independence (*p* for non-linearity = 0.024), suggesting that its predictive value does not increase uniformly across the entire score range.

**Conclusion:**

The 24-h NIHSS is a reliable early surrogate for 90-day functional recovery post-MT in both ACI and PCI, outperforming both baseline NIHSS and *Δ*-NIHSS. The non-linear relationship between 24-h NIHSS and functional independence highlights that treating it as a continuous variable has limitations, strongly supporting the use of this pragmatic cutoff rather than treating the scale as a simple continuous linear predictor.

## Introduction

Mechanical thrombectomy (MT) is the standard treatment for acute ischemic stroke due to large-vessel occlusion (1, 2). Accurately predicting functional outcome early after the procedure is crucial for clinical management and research trial design (3, 4). While the 90-day mRS is the conventional endpoint, it requires lengthy follow-up and large sample sizes, motivating reliable early surrogates (5–7).

The National Institutes of Health Stroke Scale (NIHSS) at 24 h post-MT has emerged as a promising candidate surrogate for the 90-day mRS. Post-hoc analyses of randomized trials have shown its potential predictive validity, primarily in anterior circulation infarctions (ACI) (6, 7). However, these findings originated from post-hoc analyses of randomized controlled trials (RCTs), limiting their generalizability to real-world settings (6, 7). Moreover, the applicability of these predominantly Western findings to Asian populations, which carry a disproportionately high stroke burden, remains unverified (8). The NIHSS may have a nonlinear association with clinical outcomes (9), it remains unconfirmed whether this nonlinearity applies to 90-day favorable functional outcome. The NIHSS has limitations in evaluating PCI (10). A critical unanswered question is whether its prognostic validity differs significantly between ACI and PCI. However, no research has explored the interaction between infarct location (ACI or PCI) and NIHSS scores in predicting favorable 90-day functional outcomes.

To address these specific gaps, we conducted a two-center, prospective cohort study. Our primary aim was to evaluate and validate the 24-h NIHSS as a surrogate for the 90-day mRS in patients undergoing MT, with specific focus on its performance in both ACI and PCI. Secondary objectives included exploring potential non-linearity in this association and formally testing for interaction effects based on infarct location.

## Method

### Study design and participants

This was a prospective observational cohort study conducted at two comprehensive stroke centers. Both centers adopted unified treatment protocols in accordance with contemporary Chinese stroke guidelines, and all data were prospectively collected and assessed by neurologists using standardized case report forms. From June 2015 to June 2022, we consecutively recruited acute ischemic stroke (AIS) patients who underwent MT from two hospitals located in western (Department of Neurology of the First Affiliated Hospital of Chengdu Medical College) and eastern (Department of Neurology of Nanjing First Hospital) China, respectively. This study was conducted in accordance with the ethical principles of the Declaration of Helsinki and the International Conference on Harmonisation Good Clinical Practice (ICH-GCP) guidelines. The study protocol and the use of the prospective stroke registry data for this analysis were reviewed and approved by the First Affiliated Hospital of Chengdu Medical College and Nanjing First Hospital. Written informed consent was obtained from all participating patients or their legally authorized representatives prior to data collection for the registry.

The patient inclusion criteria were as follows (according to Population, Intervention, Comparison, Outcomes, and Study design principles[PICOS] principles): (1) age ≥18 years; (2) clinical diagnosis of acute ischemic stroke with a large-vessel occlusion confirmed by digital subtraction angiography; (3) treatment with MT within 6 h of symptom onset; (4) baseline brain CT or MRI ruling out intracranial hemorrhage. The exclusion criteria were as follows: (1) history of intracranial hemorrhage within the preceding 3 months; (2) known bleeding diathesis (e.g., platelet count <100 × 10^9/L or international normalized ratio >1.7); (3) known allergy to iodinated contrast media or medications required for the procedure; (4) blood glucose < 2.8 or > 22.0 mmol/L.

### Data collection

All participants underwent baseline assessments by neurologists and follow-up evaluations at 90 days post-enrollment. Patient data will be collected using standardized forms. At baseline, we will document demographic information (e.g., gender, age), clinical characteristics (e.g., stroke subtype), medical history (e.g., hypertension), biochemical test results (e.g., blood glucose), and NIHSS scores (assessed at baseline and 24 h post-MT). The NIHSS is a validated instrument for quantifying stroke severity across neurological domains, where 0 indicates normal/near-normal function and higher scores reflect greater impairment. NIHSS assessments will be performed by neurologists with over 3 years of clinical experience. At 90-day follow-up, functional outcomes (mRS) and survival status were assessed by certified neurologists through structured face-to-face or telephone interviews. Scores on the mRS range from 0 to 6, with 0 indicating no symptoms, 1 symptoms without clinically significant disability, 2 slight disability, 3 moderate disability, 4 moderate-to-severe disability, 5 severe disability, and 6 death.

### Variable definitions

The primary outcome was favorable functional status, which was defined as a mRS score of 0 to 2 at 90 days after the procedure. The baseline NIHSS was defined as the NIHSS score recorded at hospital admission prior to thrombectomy. The 24-h NIHSS was defined as the NIHSS score assessed at 24 (±2) hours after the end of the thrombectomy procedure. And the ΔNIHSS was defined as the percentage reduction in NIHSS score, calculated as ([baseline NIHSS score −24-h NIHSS]/baseline NIHSS score × 100%).

Reperfusion status was estimated after MT using the expanded Treatment in Cerebral Ischemia scale, and a grade ≥2b was defined as successful reperfusion (11). Stroke etiology was estimated using the Trial of ORG 10172 in Acute Stroke Treatment (TOAST) criteria as follows: (1) large-artery atherosclerosis, (2) cardioembolism, and (3) other or undetermined etiology of stroke (12). Hemorrhagic transformation was defined as any evidence of intracranial hemorrhage at the site of MT on imaging (13).

### Statistical analysis

In this study, continuous variables were presented as the mean (standard deviation, SD) or the median (interquartile range, IQR), depending on whether the data conformed to a normal distribution. Normality was assessed using the Shapiro–Wilk test. Categorical variables were presented as percentages.

Our analyses were conducted as follows: First, baseline characteristics of the included population were systematically summarized. To identify potential confounders that might differentially affect 90-day functional outcomes beyond the 24-h NIHSS, we compared clinical characteristics between the ACI and PCI subgroups. Second, univariate analyses were performed using the χ^2^ test and Mann–Whitney U test for primary outcomes. Variables demonstrating statistical significance in unadjusted univariate analyses (*p* < 0.05), together with NIHSS scores (baseline NIHSS, 24-h NIHSS, and ΔNIHSS), were entered into multivariate logistic regression analysis to identify independent predictors of functional outcome after MT; Third, the receiver operating characteristic (ROC) curve was used to determine the optimal cutoff value of the NIHSS score for predicting a favorable outcome at 90 days. With reference to previous literature, the NIHSS score was transformed into a 7-level ordinal variable (<5, 5–9, 10–14, 15–19, 20–24, 25–41, and 42) (6), and its association with the 90-day functional independence was assessed using logistic regression. We further evaluated the optimal predictive NIHSS score (baseline NIHSS, 24-h NIHSS, and ΔNIHSS) for 90-day functional independence, which was assessed using AUC (area under the receiver operating characteristic curve) and AIC (Akaike information criterion) values (14). To assess the interaction between infarct location (ACI/PCI) and the NIHSS metrics in predicting the primary outcome, we incorporated an interaction term into the multivariate logistic regression model. Specifically, the model included the main effects (infarct location and the NIHSS score) as well as their multiplicative interaction term (infarct location × NIHSS score). Multicollinearity among the final model variables was assessed using variance inflation factors (VIF), with a VIF > 5 indicating significant collinearity. Finally, restricted cubic splines and median reference values were used to evaluate the association shape between the optimal predictive factors among NIHSS scores as continuous variables and functional independence.

Due to the low missing rate (3.2%) for key data, we did not perform imputation but instead censored (i.e., deleted) the missing data. According to the suggestions, we retained three significant figures for the odd ratio (OR) values (15). A two-sided *p* < 0.05 threshold was used to define statistical significance. All statistical analyses were performed using Stata software (version 18; Stata Corp, College Station, TX) and R software (version 4.4.1).

## Results

### Baseline characteristics

Of the 374 patients initially enrolled in the registry, 12 (3.2%) were excluded due to missing key NIHSS scores, and a further 28 (7.5%) were lost to 90-day follow-up. Consequently, 334 patients (89.3%) of the original cohort constituted the final analysis population. The patient flow is detailed in Supplementary Figure 1. The median age of the 334 participants was 73 (64–80) years, including 198 (59.3%) males. The baseline NIHSS score and 24-h NIHSS were 16 (11–19) and 14 (6–20), respectively. Among the included patients, 282 (84.4%) had ACI and 52 (15.6%) had PCI. Detailed baseline characteristics are presented in Table 1. Patients in the PCI group were significantly younger than those in the ACI group (median age, 68 [IQR, 59–77] vs. 74 [64–81] years; *p* = 0.010) (see Table 1). However, they presented with more severe neurological deficits, as evidenced by higher NIHSS scores at admission (median, 18 [IQR, 11–29] vs. 15 [11-19]; *p* = 0.024) and 24 h post-MT (median, 20 [IQR, 6–29] vs. 14 [6–18]; *p* = 0.017) (see Table 1). In contrast, the prevalence of chronic conditions including hypertension (67.3% vs. 61.6%; *p* = 0.432) and diabetes (26.9% vs. 19.2%; *p* = 0.201) was comparable between the two groups (see Table 1).

**Table 1:** Baseline characteristics.

	All patients (334)	Anterior circulation infarction (282)	Posterior circulation infarction (52)	*p*-value^*^
Age (years)	73 (64–80)	74 (64–81)	68 (59–77)	0.010
Male (%)	198/334 (59.3)	159/282 (56.4)	39/52 (75.0)	0.012
Hypertension (%)	208/333 (62.5)	173/281 (61.6)	35/52 (67.3)	0.432
Diabetes (%)	68/334 (20.4)	54/282 (19.2)	14/52 (26.9)	0.201
Coronary artery disease (%)	60/334 (18.0)	49/282 (17.4)	11/52 (21.2)	0.514
Atrial fibrillation (%)	116/334 (34.7)	102/282 (36.5)	13/52 (25.0)	0.109
Previous stroke (%)	72/334 (21.6)	62/282 (22.0)	10/52 (19.2)	0.657
Pre-antiplatelet (%)	61/334 (18.3)	54/282 (19.2)	7/52 (13.5)	0.329
Pre-statin (%)	50/334 (15.0)	45/282 (16.0)	5/52 (9.6)	0.239
Smoking (%)	125/334 (37.4)	99/282 (35.1)	26/52 (50.0)	0.041
Systolic Blood Pressure on admission (IQR)	140 (125–155)	140 (123–155)	142 (128–160)	0.492
Diastolic Blood Pressure on admission (IQR)	83 (73–94)	83 (73–94)	83 (74–95)	0.991
NIHSS score on admission (IQR)	16 (11–19)	15 (11–19)	18 (11–29)	0.024
Fasting glucose (IQR)	6.8 (5.4–8.4)	6.8 (5.4–8.6)	7.0 (5.7–8.1)	0.500
Glycosylated hemoglobin (IQR)	6.0 (5.6–6.3)	6.0 (5.6–6.4)	6.0 (5.7–6.3)	0.921
TOAST classification (%)				<0.001
Large-artery atherosclerosis	118/334 (35.3)	86/282 (30.5)	32/53 (61.5)	
Cardio embolism	169/334 (50.6)	153/282 (54.3)	16/52 (30.8)	
Others	47/334 (14.1)	43/282 (15.3)	4/52 (7.7)	
Treatment with IV tPA	122/334 (36.5)	110/282 (39.0)	12/53 (23.1)	0.028
OTT min (IQR)	300 (210–360)	289 (200–350)	319 (240–463)	0.047
mTICI 2b-3 (%)	288/334 (86.2)	245/282 (86.9)	43/52 (82.7)	0.421
24-h NIHSS (IQR)	14 (6–20)	14 (6–18)	20 (6–29)	0.017
Hemorrhagic transformation (%)	65/334 (19.5)	55/282 (19.5)	10/52 (19.2)	0.964

* Differences in baseline characteristics between patients with anterior and posterior circulation infarction were assessed using the Chi-squared test for categorical variables and the Wilcoxon rank-sum test for continuous variables; TOAST trial of Org 10,172 in acute stroke treatment; NIHSS National Institute of Health stroke Scale; OTT onset treatment time.

### Univariate and multivariate analyses

Univariate analysis identified several factors that differed significantly between patients with favorable and unfavorable 90-day outcomes. These included, in addition to baseline and 24-h NIHSS scores, age, sex, smoking status, fasting blood glucose, successful vascular recanalization rate, and the occurrence of hemorrhagic transformation (Table 2).

**Table 2:** Univariate analysis of 90-day favorable function outcome.

	mRS 0–2 (*n* = 117)	MRS 3–6 (*n* = 217)	*p*-value
Age (years) (IQR)	68 (60–77)	75 (66–82)	<0.001
Male gender (%)	81 (69.2)	117 (53.9)	0.007
Hypertension (%)	68 (58.1)	140 (64.5)	0.250
Diabetes (%)	21 (18.0)	47 (21.7)	0.422
Smoking (%)	60 (51.3)	65 (30.0)	<0.001
Coronary artery disease (%)	22 (18.8)	38 (17.5)	0.769
Hyperlipidemia (%)	4 (3.4)	5 (2.3)	0.548
Atrial fibrillation (%)	40 (34.2)	76 (35.0)	0.879
Previous stroke (%)	19 (16.2)	53 (24.4)	0.083
Pre-antiplatelet (%)	19 (16.2)	42 (19.4)	0.482
Pre-statin (%)	16 (13.7)	34 (15.7)	0.626
Total cholesterol (IQR)	4.0 (3.4–5.0)	4.1 (3.5–5.0)	0.830
SBP(IQR)	143 (126–152)	140 (123–157)	0.862
DBP(IQR)	82 (73–93)	83 (73–95)	0.247
LDL(IQR)	2.3 (2.0–3.0)	2.3 (2.0–3.0)	0.573
Triglyceride (IQR)	1.0 (0.8–1.5)	1.0 (0.8–1.4)	0.693
NIHSS score on admission (IQR)	13 (12–18)	16 (13–21)	<0.001
Fasting glucose (IQR)	6.0 (5.0–7.6)	7.1 (5.8–9.0)	<0.001
Glycosylated hemoglobin (IQR)	5.9 (5.5–6.3)	6.0 (5.6–6.5)	0.262
Platelet count (IQR)	175 (135–220)	169 (135–206)	0.377
INR(IQR)	1.00 (0.95–1.03)	1.00 (0.96–1.04)	0.340
TOAST classification (%)			0.116
LAA	49 (41.9)	69 (31.8)	
CE	56 (47.9)	113 (52.1)	
Other all	12 (10.3)	35 (16.1)	
Treatment with IV tPA (%)	45 (38.5)	77 (35.5)	0.590
OTT min (IQR)	300 (221–357)	287 (200–360)	0.260
Occlusion site (%)			0.701
Anterior circulation	100 (85.5)	182 (83.9)	
Posterior circulation	17 (14.5)	2 (16.1)	
mTICI 2b-3 (%)	109 (93.2)	179 (82.5)	0.007
24-h NIHSS (IQR)	5 (3–12)	16 (12–23)	<0.001
Hemorrhagic transformation (%)	11 (9.4)	54 (24.9)	0.001

TOAST (trial of Org 10,172 in acute stroke treatment); LAA (large-artery atherosclerosis); CE (cardio embolism); IQR (interquartile range); INR (International Normalized Ratio); NIHSS (National Institute of Health stroke Scale); OTT (onset treatment time); LDL (Low Density Lipoprotein).

Multivariate logistic regression analysis, adjusted for age, sex, smoking status, fasting blood glucose, successful recanalization, and hemorrhagic transformation, confirmed that lower baseline NIHSS score (OR 1.73, 95% Confidence Interval [CI] 1.42–2.12, *p* < 0.001), 24-h NIHSS (OR 2.73, 95%CI 2.19–3.40, *p* < 0.001), and ΔNIHSS score (OR 1.96, 95%CI 1.68–2.28, *p* < 0.001) were all significantly associated with functional independence at 90 days (see Table 3). Among them, lower 24-h NIHSSs had a stronger association with functional independence.

**Table 3:** Association of various thresholds of NIHSS with 90-day modified Rankin Scale 0–2.

Group	Definitions	Unadjusted odds ratio	*p* value	Adjusted odds ratio^*^	*p* value	VIF Range	Sensitivity	Specificity	AIC	Cut-off values	*p* for interaction^ǂ^
All infarctions											
Baseline NIHSS score		1.73 (1.42–2.12)^$^	<0.001	1.54 (1.25–1.90)^$^	<0.001	1.03-1.57	77.9%	49.6%	410.7	12	0.340
24-h NIHSS score after MT		2.73 (2.19–3.40)^$^	<0.001	2.53 (2.00–3.20)^$^	<0.001	1.07-1.55	94.9%	65.8%	288.7	6	0.741
Percent change in NIHSS^#^		1.96 (1.68–2.28)^&^	<0.001	1.92 (1.62–2.28)^&^	<0.001	1.06-1.59	74.2%	75.2%	358.8	8%	0.467
Anterior circulation infarction											
Baseline NIHSS score		1.66 (1.31–2.09)^$^	<0.001	1.40 (1.09–1.81)^$^	0.009	1.03-1.57	83.0%	40.0%	360.5	12	/
24-h NIHSS score after MT		2.75 (2.15–3.51)^$^	<0.001	2.56 (1.96–3.35)^$^	<0.001	1.05-1.57	95.1%	65.0%	247.3	6	/
Percent change in NIHSS^#^		2.00 (1.68–2.38)^&^	<0.001	1.98 (1.63–2.40)^&^	<0.001	1.06-1.59	73.1%	77.0%	310.2	8%	/
Posterior circulation infarction											
Baseline NIHSS score		1.99 (1.30–3.04)^$^	0.002	1.85 (1.13–3.03)^$^	0.014	1.09-1.60	80.0%	64.7%	59.7	14	/
24-h NIHSS score after MT		2.62 (1.62–4.25)^$^	<0.001	3.13 (1.46–6.70)^$^	0.003	1.13-1.66	77.1%	94.1%	43.2	18	/
Percent change in NIHSS^#^		1.78 (1.26–2.51)^&^	0.001	1.81 (1.18–2.76)^&^	0.006	1.11-1.42	82.9%	64.7%	62.0	18%	/

* The regression model, based on the categorized NIHSS score, included age, gender, smoking, rapid blood glucose, mTICI 2b - 3, and hemorrhagic transformation.

ǂ The interaction term was defined as NIHSS score (categorical) × infarct location (anterior circulation infarction or posterior circulation infarction).

$ The NIHSS scores were categorized into a 7-level ordinal variable using the following thresholds: <5, 5–9, 10–14, 15–19, 20–24, 25–41, and 42.

# Percent change in NIHSS calculated as a threshold of change in NIHSS within 24 h of EVT/baseline NIHSS×100.

& This continuous variable was subsequently transformed into a 7-level ordinal variable based on its septiles.

NIHSS, National Institute of Health stroke Scale; AIC, Akaike information criterion; VIF, Variance Inflation Factor.

### Predictive performance of NIHSS metrics

**Figure 1:**
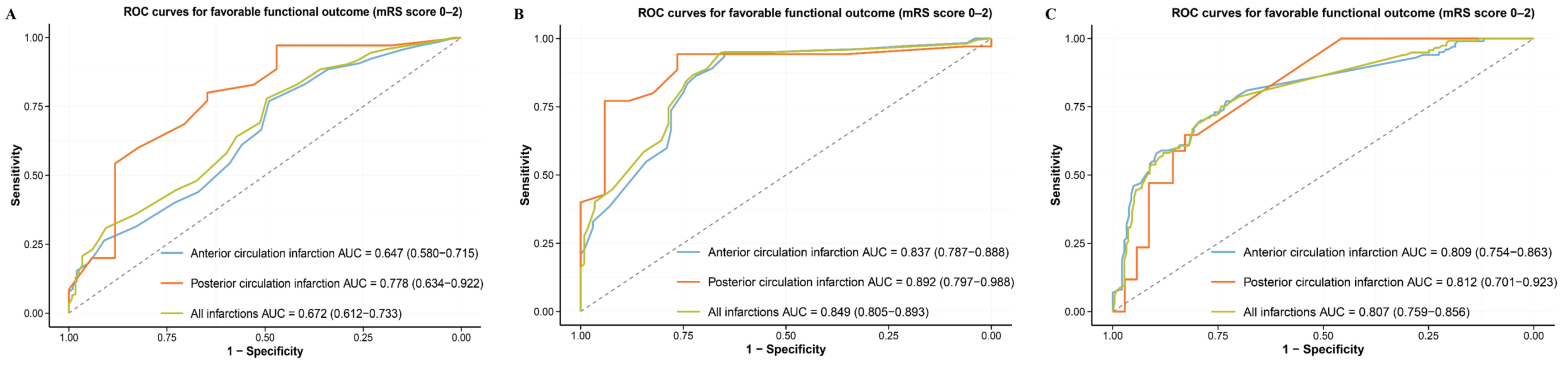
Receiver operating characteristics of National Institutes of Health Stroke Scale (NIHSS)-based outcome measures at predicting 90-d modified Rankin Score (mRS) 0–2. **(A)** 24-hours NIHSS, **(B)** NIHSS (baseline mines 24-hours NIHSS), and **(C)** percent change in NIHSS ([baseline NIHSS - 24-hours NIHSS]/baseline NIHSS×100).

The 24-h NIHSS demonstrated the highest discriminative ability for 90-day functional independence, with an AUC of 0.849 (95% CI: 0.805–0.893), superior to both baseline NIHSS (AUC: 0.672, 95% CI: 0.612–0.733) and ΔNIHSS (AUC: 0.807, 95% CI: 0.759–0.856) (Figure 1). Its optimal cutoff, determined by maximizing the Youden index, was ≤6, yielding a sensitivity of 94.9% and a specificity of 65.8%. The corresponding cutoffs, sensitivity, and specificity for baseline NIHSS and ΔNIHSS are provided in Table 3. The model fit was best for the 24-h NIHSS, as evidenced by its substantially lower AIC value (288.7) compared to models containing baseline NIHSS (410.7) or ΔNIHSS (358.8) (Table 3). Collectively, these results establish the 24-h NIHSS as the optimal predictor.

### Non-linear relationship between 24-h NIHSS and 90-day favorable functional

**Figure 2:**
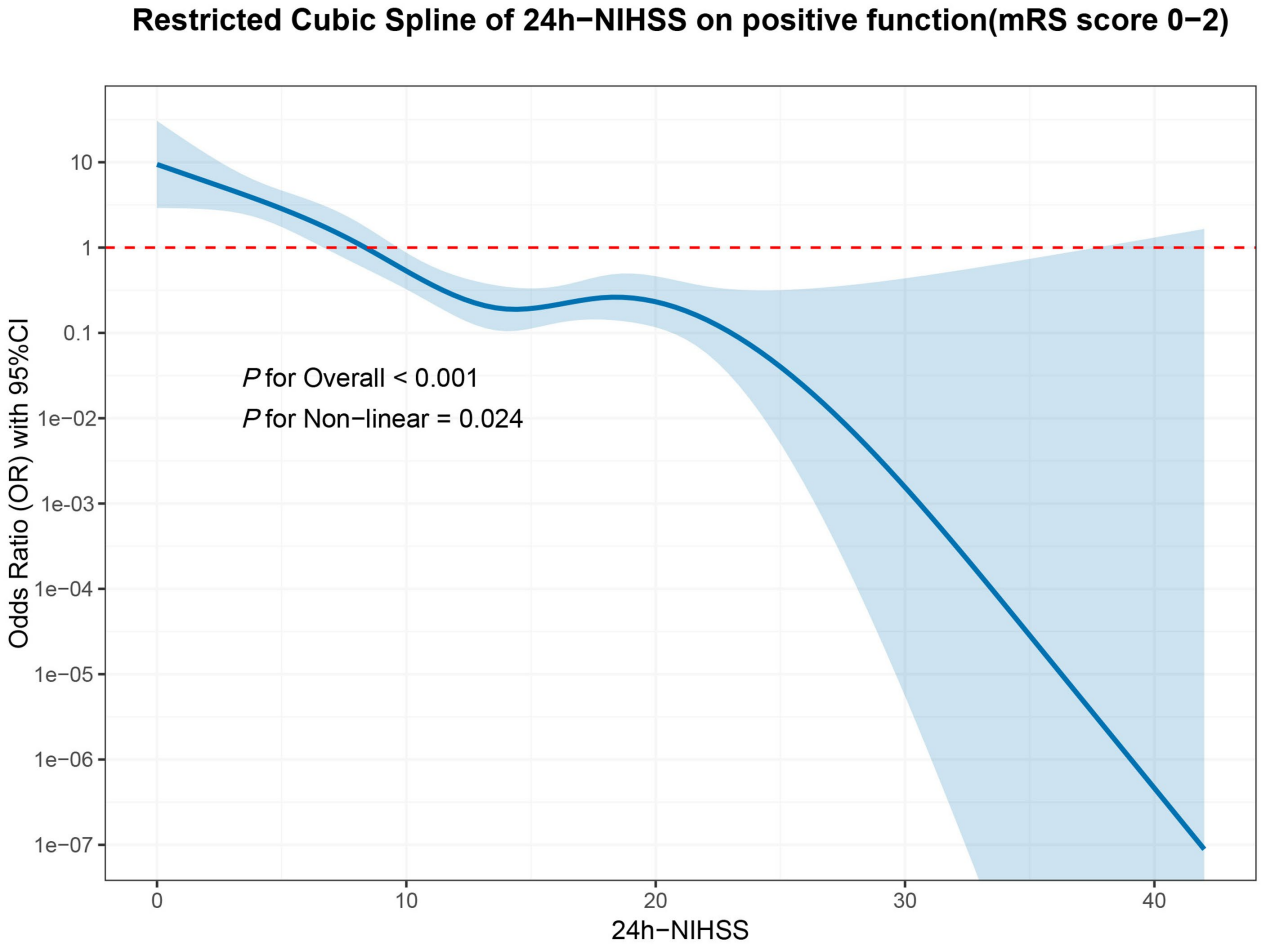
Nonlinear prediction of 90-day modified Rankin Scale score based on the 24-hours National Institutes of Health Stroke Scale (NIHSS) score. In this linear prediction model, 24-hours NIHSS is treated as a nonlinear variable. Upper and lower bounds of this prediction are shown with the gray area plot.

The functional form of the association between the continuous 24-h NIHSS and the log-odds of 90-day functional independence was modeled using a restricted cubic spline (RCS) with 3 knots placed at default percentiles. The association was statistically significant (*p* for overall < 0.001) and exhibited clear non-linearity (*p* for non-linearity = 0.024), as shown in Figure 2.

The RCS analysis (Figure 2) showed a plateau in the curve between scores of 12 and 19, where each additional point on the 24-h NIHSS was associated with a progressively smaller reduction in the likelihood of functional independence. This non-linear relationship provides statistical justification for moving beyond a linear interpretation of the 24-h NIHSS score in clinical practice.

### Subgroup analysis

In the ACI cohort and PCI cohort, 24-h NIHSS scores were the best predictors of 90-day functional independence (see Figure 1; Table 3). The areas under the AUC values were 0.837 (0.787–0.888) and 0.892 (0.797–0.988), respectively. Moreover, for baseline NIHSS, 24-h NIHSS, and ΔNIHSS scores, the AUC values of PCI was higher than that of ACI and all patients. Formal testing found no significant interaction between any NIHSS metric and infarct location (ACI vs. PCI) in predicting the outcome (all *p* for interaction > 0.05, Table 3), indicating its prognostic validity did not differ by infarction location.

## Discussion

This study, based on a cerebral infarction cohort including both ACI and PCI, demonstrates that the 24-h NIHSS is the strongest predictor of 90-day functional independence after MT. Moreover, the association between 24-h-NIHSS and 90 - day functional independence was non - linear. To our knowledge, this study provides the first direct evidence confirming that there was no interaction between NIHSS scores and infarction location (ACI/PCI) in predicting the prognosis of patients after MT.

Our findings regarding the predictive superiority of the 24-h NIHSS are consistent with the work of Ospel et al., who validated its utility as a surrogate endpoint in RCTs (6). However, our study extends this evidence by demonstrating its robust prognostic value in a real-world, prospective cohort that included both ACI and PCI. The optimal cutoff in our study, identified by maximizing the Youden index, was 6 for the 24-h NIHSS. This value is consistent with the range (5–7) reported in prior studies that exclusively enrolled either ACI or PCI patients (16, 17). Clinical data further indicate that dichotomizing the 24-h NIHSS at this threshold achieves an 80.25% agreement with the 90-day mRS (18). The NIHSS score of ≤6, which indicates minimal to mild neurological impairment, aligns with the clinical objective of functional independence (mRS ≤ 2), thereby reinforcing the relevance of this cutoff. Furthermore, unlike prior studies focused on surrogate endpoint, we identified a significant non-linear relationship, suggesting caution when using it as a simple continuous variable for outcome prediction.

The cumulative evidence from this study suggests that the 24-h NIHSS is the strongest predictor of prognosis in cerebral infarction patients after MT. This is because the 24-h NIHSS, as an early measure of neurological status, demonstrates a stronger association with functional outcomes in MT-treated cerebral infarction patients than the baseline NIHSS score (OR 0.83 [0.81–0.84] vs. OR 0.96 [0.95–0.96]) (19). Mediation analysis revealed that the 24-h NIHSS explained 54% of the variance in 90-day functional independence (19). This implies that clinicians may be able to identify high-risk patients within 24 h.

Recently, studies have recommended using the 24-h NIHSS as the primary outcome in acute ischemic stroke trials to replace the 90-day mRS score, owing to its high consistency with functional outcomes and shorter follow-up period (6, 7). However, we must recognize the limitations of the 24-h NIHSS. Compared with the mRS, the NIHSS lacks assessment of cognitive impairment and functional disability in patients (6). Between 24 h and 90 days after MT, cerebral infarction patients may develop severe complications (such as pneumonia) that affect prognosis (6). Therefore, when using the 24-h NIHSS as the primary outcome, these confounding factors need to be considered. It is also important to note the skewed distribution of the 24-h NIHSS and its non-linear relationship with 90-day functional independence.

Our study found a non-linear association between the 24-h NIHSS and 90-day favorable function (*p* for non-linearity = 0.024), which implies that using the 24-h NIHSS as a continuous variable for primary outcomes may have limitations. Consequently, treating the 24-h NIHSS as a simple continuous linear variable in analysis is statistically suboptimal. This non-linearity strongly supports transforming it into an ordinal variable, as previously done in other studies, thereby better reflecting its predictive nature (6, 20). Although the 24-h NIHSS has certain limitations as a primary outcome, it retains value in specific clinical scenarios, such as the early assessment of functional recovery after MT and the prediction of futile recanalization. When these factors are appropriately considered, particularly by using categorical rather than continuous transformations, the 24-h NIHSS can serve as a valuable and efficient prognostic tool. Our results support its suitability as a rapid clinical indicator for MT efficacy, a conclusion that merits further validation in future studies.

In the subgroup analysis, we found no interaction between NIHSS scores in ACI and PCI groups. In our cohort, 84.4% of patients had ACI and 15.6% had PCI. This proportion is similar to a previous study involving 552 cerebral infarction patients treated with MT, which reported 85% ACI and 13.2% PCI (21). The natural cohort of real-world data ensures high external validity and generalizability of our findings (22). A pivotal finding of our study is the lack of a significant interaction between NIHSS metrics and infarct location. This consistency is clinically reassuring, particularly considering the known limitations of the NIHSS in fully capturing posterior circulation deficits (e.g., truncal ataxia, complex cranial nerve palsies) (23). It suggests the 24-h NIHSS is a robust predictor of global functional outcome valid for both stroke subtypes. However, we observed different optimal cutoff values for the two territories (6 in ACI vs. 18 in PCI). This discrepancy does not contradict the absence of an interaction. This discrepancy most likely originates from a difference in the distribution of 24-h NIHSS scores between the PCI and ACI populations (24). Specifically, in our cohort, patients with PCI presented with a higher median 24-h NIHSS score. In practical terms, this means that while the same NIHSS score carries a similar relative predictive value in both groups, the absolute risk at a given score differs. Consequently, for risk stratification in clinical practice, applying a territory-specific cutoff may be more appropriate than a single universal threshold, even though the underlying predictive model is generalizable.

In the sensitivity analysis, we demonstrated that regression results were consistent both before and after adjusting for confounding factors. This indicates that our findings are robust.

The strengths of this study include its use of real-world data from non-developed countries in East Asia, which enhances the generalizability of the findings. Unlike previous studies, our research included both ACI and PCI patients, and further explored the interaction between cerebral infarction location and NIHSS scores on the prognosis of patients after MT. Our results address clinicians’ uncertainties regarding the differences in assessment efficacy of NIHSS scores between ACI and PCI. Additionally, the non-linear relationship between 24-h NIHSSs and 90-day functional independence highlights the limitations of using 24-h NIHSS as a primary outcome measure.

It is important to acknowledge the limitations of this study. First, the sample size of PCI patients in our cohort was small. Although this reflects the real-world proportion in clinical practice, it may lead to imprecision in the results of our PCI subgroup analysis. Second, as an observational study, we can only attempt to exclude rather than completely avoid confounding factors, and our findings require validation by higher-quality studies. Third, while our data were collected from two advanced hospitals in eastern and western China, the risk of selection bias remains, and our findings may not be generalizable to regions with lower healthcare resources or standards. Fourth, the exclusion of patients who received MT beyond 6 h of onset, or those with a history of intracranial hemorrhage within 3 months or abnormal glucose levels, may limit the generalizability of our findings to these specific populations. Fifth, our study lacked advanced imaging variables (such as infarct core volume) and detailed reperfusion metrics. These omissions may affect the assessment of confounding factors and the precision of the predictive model. Six, the absence of data on in-hospital complications (e.g., pneumonia, DVT) limits our ability to fully account for their confounding effects on 90-day outcomes. Future studies should prioritize incorporating these important confounding factors.

In conclusion, our study findings establish the 24-h NIHSS as the strongest predictor of 90-day functional independence in post-MT patients with either ACI or PCI, making it suitable as a primary indicator for early assessment of MT efficacy. Moreover, the identified non-linear relationship justifies employing it as a categorical, rather than continuous, measure.

## Data Availability

The raw data supporting the conclusions of this article will be made available by the authors, without undue reservation.

## References

[1] Turc G Bhogal P Fischer U Khatri P Lobotesis K Mazighi M . European stroke organisation (ESO) - European Society for Minimally Invasive Neurological Therapy (ESMINT) guidelines on mechanical thrombectomy in acute ischemic stroke. J Neurointerv Surg. (2023) 15:e8. doi: 10.1136/neurintsurg-2018-014569, 30808653

[2] Amoukhteh M Hassankhani A Ghozy S Valizadeh P Jannatdoust P Bilgin C . Mechanical Thrombectomy for in-hospital onset stroke: a comparative systematic review and meta-analysis. J Stroke. (2024) 26:41–53. doi: 10.5853/jos.2023.01613, 38186183 PMC10850456

[3] Rudilosso S Laredo C Amaro S Renú A Llull L Obach V . Clinical improvement within 24 hours from mechanical thrombectomy as a predictor of long-term functional outcome in a multicenter population-based cohort of patients with ischemic stroke. J Neurointerv Surg. (2021) 13:119–23. doi: 10.1136/neurintsurg-2020-015934, 32461229

[4] Kleine JF Boeckh-Behrens T Prothmann S Zimmer C Liebig T. Discrepancy between early neurological course and mid-term outcome in older stroke patients after mechanical thrombectomy. J Neurointerv Surg. (2016) 8:671–6. doi: 10.1136/neurintsurg-2015-011702, 26047902

[5] Rethnam V Bernhardt J Johns H Hayward KS Collier JM Ellery F . Look closer: the multidimensional patterns of post-stroke burden behind the modified Rankin scale. Int J Stroke. (2021) 16:420–8. doi: 10.1177/1747493020951941, 32854602

[6] Ospel JM Brown S Bosshart S Stebner A Uchida K Demchuk A . Modified Rankin scale at 90 days versus National Institutes of Health stroke scale at 24 hours as primary outcome in acute stroke trials. J Am Heart Assoc. (2025) 14:e037752. doi: 10.1161/jaha.124.037752, 39968801 PMC12132765

[7] Stebner A Bosshart SL Demchuk A Poppe A Nogueira R McTaggart R . Factors influencing the association of 24-hour National Institutes of Health stroke scale & 90-day modified Rankin score. Clin Neuroradiol. (2025) 35:141–50. doi: 10.1007/s00062-024-01459-3, 39404848

[8] Datta A Akundi S Wagh K Bhurle G Sarmah D Sharma A . Stroke and associated comorbidities in southeast Asian countries. Neuroprotection. (2025) 3:29–47. doi: 10.1002/nep3.71, 41383526 PMC12486952

[9] Elsaid AF Fahmi RM Shehta N Ramadan BM. Machine learning approach for hemorrhagic transformation prediction: capturing predictors' interaction. Front Neurol. (2022) 13:951401. doi: 10.3389/fneur.2022.951401, 36504664 PMC9731336

[10] Siniscalchi A Sztajzel R Malferrari G Gallelli L. The National Institutes of Health stroke scale: its role in patients with posterior circulation stroke. Hosp Top. (2017) 95:79–81. doi: 10.1080/00185868.2017.1322888, 28535100

[11] Goyal M Fargen KM Turk AS Mocco J Liebeskind DS Frei D . 2C or not 2C: defining an improved revascularization grading scale and the need for standardization of angiography outcomes in stroke trials. J Neurointerv Surg. (2014) 6:83–6. doi: 10.1136/neurintsurg-2013-010665, 23390038 PMC4156591

[12] Adams HP Jr Bendixen BH Kappelle LJ Biller J Love BB Gordon DL . Classification of subtype of acute ischemic stroke. Definitions for use in a multicenter clinical trial. TOAST. trial of org 10172 in acute stroke treatment. Stroke. (1993) 24:35–41. doi: 10.1161/01.str.24.1.35, 7678184

[13] Fiorelli M Bastianello S von Kummer R del Zoppo GJ Larrue V Lesaffre E . Hemorrhagic transformation within 36 hours of a cerebral infarct: relationships with early clinical deterioration and 3-month outcome in the European cooperative acute stroke study I (ECASS I) cohort. Stroke. (1999) 30:2280–4. doi: 10.1161/01.str.30.11.2280, 10548658

[14] Portet S. A primer on model selection using the Akaike information criterion. Infect Dis Model. (2020) 5:111–28. doi: 10.1016/j.idm.2019.12.010, 31956740 PMC6962709

[15] Cole TJ. Too many digits: the presentation of numerical data. Arch Dis Child. (2015) 100:608–9. doi: 10.1136/archdischild-2014-307149, 25877157 PMC4483789

[16] Mistry EA Yeatts S de Havenon A Mehta T Arora N De Los Rios La Rosa F . Predicting 90-day outcome after thrombectomy: baseline-adjusted 24-hour NIHSS is more powerful than NIHSS score change. Stroke. (2021) 52:2547–53. doi: 10.1161/strokeaha.120.032487, 34000830 PMC11261999

[17] Kniep H Bechstein M Broocks G Brekenfeld C Flottmann F van Horn N . Early surrogates of outcome after thrombectomy in posterior circulation stroke. Eur J Neurol. (2022) 29:3296–306. doi: 10.1111/ene.15519, 35933692

[18] Rinkel LA Ospel JM Kappelhof M Sehgal A McDonough RV Tymianski M . Comparing early National Institutes of Health stroke scale versus 90-day modified Rankin scale outcomes in acute ischemic stroke trials: a systematic review and analysis. J Am Heart Assoc. (2025) 14:e040304. doi: 10.1161/jaha.124.040304, 40281657 PMC12184254

[19] Kniep H Meyer L Bechstein M Broocks G Guerreiro H van Horn N . How much of the Thrombectomy related improvement in functional outcome is already apparent at 24 hours and at hospital discharge? Stroke. (2022) 53:2828–37. doi: 10.1161/strokeaha.121.037888, 35549377

[20] Saver JL Gornbein J Starkman S. Graphic reanalysis of the two NINDS-tPA trials confirms substantial treatment benefit. Stroke. (2010) 41:2381–90. doi: 10.1161/strokeaha.110.583807, 20829518 PMC2949055

[21] Neumann A Schildhauer P Weiler SM Schramm P Schacht H Royl G . Mechanical thrombectomy failure in anterior and posterior circulation stroke: current results from a high-volume comprehensive center. Neurol Sci. (2025) 46:807–17. doi: 10.1007/s10072-024-07881-2, 39578333 PMC11772395

[22] Khozin S Blumenthal GM Pazdur R. Real-world data for clinical evidence generation in oncology. J Natl Cancer Inst. (2017) 109:109. doi: 10.1093/jnci/djx187, 29059439

[23] Schneck MJ. Current stroke scales may be partly responsible for worse outcomes in posterior circulation stroke. Stroke. (2018) 49:2565–6. doi: 10.1161/strokeaha.118.023201, 30355229

[24] Kuribara T Iihoshi S Tsukagoshi E Teranishi A Kinoshita Y Sugasawa S . Thrombectomy for acute large vessel occlusion in posterior and anterior circulation: a single institutional retrospective observational study. Neuroradiology. (2022) 64:565–74. doi: 10.1007/s00234-021-02799-4, 34477913 PMC8850247

