# Supplementary figures and images for: 24-hour NIHSS may be a reliable surrogate for 90-day mRS after mechanical thrombectomy—a prospective study in anterior and posterior circulation infarctions
Source: Front Neurol. 2026 Jan 12;16:1698534. doi: 10.3389/fneur.2025.1698534 (PMC12832342; doi:10.3389/fneur.2025.1698534)

Supplementary Figure 1 Patient selection flowchart.

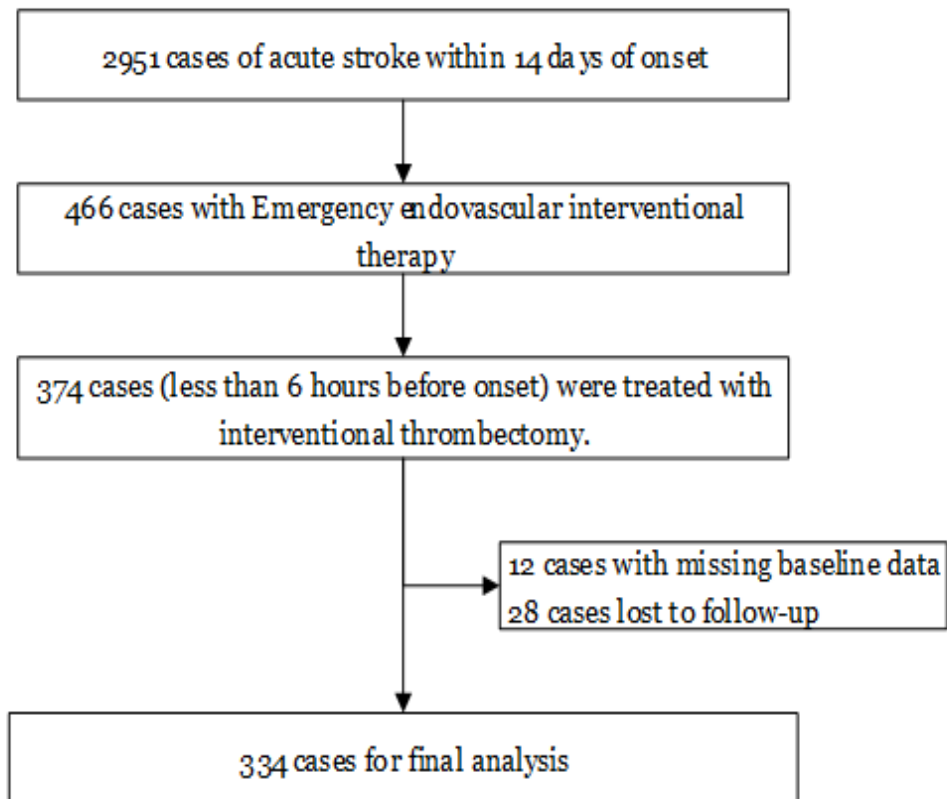

Supplement: Supplementary file 1 [file Image_1.pdf]
